# Synthesis, structural, and characterization of polymethylmethacrylate matrix−ZnO nanocomposite

**DOI:** 10.1038/s41598-025-27385-z

**Published:** 2025-12-01

**Authors:** O. N. Megahed, M. I. Abdelhamid, N. A. Elwassefy, Ahmed M. Youssef, G. El-Damrawi, N. A. Bakr

**Affiliations:** 1 Department of Physics, Faculty of Science, Mansoura University, Mansoura, Egypt; 2 Department of Dental Biomaterials, Faculty of Dentistry, Mansoura University, Mansoura, Egypt; 3 Packaging Materials Department, National Research Centre, Dokki, Giza, Egypt

**Keywords:** Polymethylmethacrylate (PMMA)–ZnO NPs, Emulsion polymerization, TGA–Vickers microhardness, Young’s modulus, Antibacterial activity, Chemistry, Materials science, Microbiology, Nanoscience and technology

## Abstract

The current work investigates the effect of incorporating zinc oxide nanoparticles (ZnO NPs) into the polymer matrix polymethylmethacrylate (PMMA), focusing on structural, thermal, mechanical, and antimicrobial properties. The synthesized polymer nanocomposites, prepared via free radical emulsion polymerization, were characterized using Fourier Transform Infrared (FTIR) spectroscopy, X-ray diffraction (XRD), transmission electron microscopy (TEM), scanning electron microscopy (SEM), thermal gravimetric analysis (TGA), and density measurements. Furthermore, the antibacterial and antimicrobial properties of the polymer nanocomposites were assessed against gram-negative bacteria (*E. coli*), gram-positive bacteria (*S. mutans*), and the fungus (*C. albicans*). Moreover, the mechanical characteristics, including Vickers micro-hardness, Young’s modulus, tensile strength, and elongation at break, for pure PMMA and PMMA/ZnO nanocomposite films have also been examined. The results confirm that the structure of the host matrix changes slightly upon the addition of ZnO NPs. The density measurement shows an increase, and the thermal stability is enhanced by ~ 6% for an optimum concentration of ZnO NPs addition. Additionally, the hardness number reaches its maximum value with an increase of ~ 13.5%, which is a statistically significant difference. Moreover, mechanical properties (e.g., Young’s modulus, tensile strength) and antibacterial and antimicrobial properties have shown remarkable changes. In conclusion, this work emphasizes optimal 3 wt% loading, attributed to the good dispersion of ZnO NPs, highlighting enhanced performance that can be used in denture-base applications.

## Introduction

Acrylic resins are used in dentistry for a variety of purposes, including denture bases, acrylic resins (non-metallic), and removable base plates^1–3^. They are easy to process, nontoxic, stable in the oral environment, color-matching, easy for patients to maintain cleanliness, inexpensive, odorless, tasteless, non-irritating, lightweight, and easy to repair after fracture. Polymethyl methacrylate (PMMA) has been clinically previously used for decades, particularly in the fields of orthodontics and prosthodontics in partial and full denture production^4,5^, due to its superior biological, physicochemical, and aesthetic qualities^4,5^. It was a low-cost transparent hydrophobic thermoplastic polymer with a porous and amorphous nature^6–8^, low ductility, and high thermal and chemical stability^6,7,9^. It has many more advantages, such as excellent biocompatibility, polishability, ease of processing in the laboratory, and no taste or smell after polymerization^4,5,10^. However, the most prevalent clinical issues with denture fracture are insufficient mechanical strength and the antimicrobial effect of PMMA. The insufficient antimicrobial effect leads to dental plaque biofilm deposition in the oral environment, resulting in dental caries and mucosal infections, limiting and restricting it as an ideal clinical use for removable prosthetic devices^1^. Thus, the most desirable feature nowadays is to reinforce PMMA by organic and inorganic substances (e.g., rods, fibers, metal wires, metal oxides, metal nets, or clays)^4,10^.

In recent years, nanoscale semiconductors such as zinc oxide nanoparticles (ZnO NPs) have been widely used as multifunctional inorganic fillers in nanocomposite reinforced materials. They have gained more attention because of their notable physical and chemical characteristics, including their high refractive index, nontoxicity, and biocompatibility, as well as effective broad-spectrum antimicrobial activity against both gram-positive and gram-negative bacteria. ZnO NPs also possess higher chemical stability and thermal conductivity^5,7,9,11^. Previous studies^12,13^ have shown that ZnO NPs have antifungal properties and are safe for usage as a new alternative biomaterial for denture base nanocomposites. Therefore, the nano ZnO particles have been a suitable filler material that could be added to polymers in various compositions to enhance both their mechanical and antimicrobial properties^1,5,7,9,11^. The methods that are used for the synthesis of such nanocomposite materials are mainly categorized into chemical and physical methods, where the latter have no control over the shape and size of the synthesized particles, shifting our interest to chemical methods^6^.

Conventional free radical emulsion polymerization was used to synthesize PMMA/ZnO nanocomposites. This method is highly effective for creating nanocomposites, where the initiating radical is generated when a pure monomer or an extra initiator is thermally or photochemically decomposed. ZnO NPs, like other nanoparticles, have a high surface energy and tend to agglomerate when dispersed in organic solvent matrices. Agglomeration can be avoided by preparing ZnO NPs before the polymerization process^11^, using the sol-gel technique. The latter is considered the most popular technique for its superior uniformity, low processing temperatures, simple composition control, and affordability^14^. Obtaining fine dispersion of ZnO NPs (without surface modification) into the polymer matrix can be achieved by using an ultrasonic tool^6^. Most of the previous studies demonstrate that ZnO NPs should be added as a dopant at the lowest concentration. This may be because the homogenous distribution at high content (~ 10 wt%) of ZnO NPs leads to immiscibility and high surface energy^7^. For this reason, this current study uses modest quantities (1–5 wt%) of the loaded ZnO NPs to avoid any aggregation into the matrix. The structure of the well-synthesized nanocomposites has changed slightly through intermediate dopant addition. However, some physical, thermal, and mechanical properties have to be changed in the direction of remarkable improvement. Therefore, this current study represents a significant development of PMMA/ZnO nanocomposites through an optimal balance between reduced filler content and good resulting characteristics, highlighting enhanced performance and usage in denture-base applications.

The outcomes of this study can be directly aligned with the objectives of the United Nations Sustainable Development Goals (UNSDGs). By enhancing the reinforcement of polymers used in various dental applications, the findings support SDG 3 (Good Health and Well-Being) through the development of more reliable and durable denture-base materials that improve long-term oral health outcomes and patient quality of life. Furthermore, the innovation in polymer composite design contributes to SDG 9 (Industry, Innovation, and Infrastructure) by promoting sustainable advancement in dental biomaterials and fostering translational potential for more clinical applications. In addition, the improved longevity of reinforced denture material reduces the frequency of replacements, thereby minimizing material waste and resource consumption, in line with SDG 12 (Responsible Consumption and Production). Collectively, this study highlights how scientific advances in dental material science can contribute to global health and sustainability agendas.

## Experimental work

### Materials

In order to prepare ZnO NPs and PMMA/ZnO nanocomposite, Zinc Chloride Dry (Extra Pure 97%, ZnCl_2_, LOBA. CHEMIE.PVT.LTD.), Absolute Ethanol (C_2_H_5_OH, 99.8%, PIOCHEM Laboratory Chemicals), Ammonium Hydroxide [NH_4_OH] (33%, pH controller), Methyl Methacrylate Monomer (C_5_H_8_O_2_ MMA, Stabilized, Hydroquinone, SDFCL, LR grade), Sodium Hydroxide Powder (NaOH, Alpha Chemika), Sodium Lauryl Sulphate Powder (SLS, C_12_H_25_NaO_4_S LOBA CHEMIE PVT.LTD., 85% Extra Pure), Potassium Persulphate Extra Pure (KPS) (K_2_S_2_O_8_, SDFCL, 98.0%), and Toluene (99.5% AR grade) were used.

### Sample preparation

#### Preparation of zinc oxide nanoparticles (ZnO NPs) by sol-gel method

Sol-gel is one of the most widely used methods for ZnO NP synthesis due to its good homogeneity, low processing temperatures, easy composition control, and low cost. In addition, by changing the experimental conditions, including concentration, reaction time, pH, and temperature, this method can monitor the morphology and grain size of the particles^14^. A calculated amount of dry zinc chloride was dissolved in distilled water with a stirring speed of 600 rpm for around 2 h at room temperature. In the next step, an aqueous solution of NH_4_OH ammonia (33%, pH controller) was applied drop by drop till the pH of the solution reached around the 9–10 range (alkaline condition). As a result of the reaction, the suspension solution changed color from clear to milky white, indicating the formation of Zn (OH)_2_. After that, the collected white precipitate was removed, centrifuged, and repeatedly washed with a combination of ethanol and distilled water several times to get rid of any ionic impurities, then dried at 80 °C. After three hours of calcination at 600 °C with a heating rate of 5 °C/min, ZnO was ground into a fine powder prior to use. The reaction was represented in the following equations:

1 $${\text{ZnC}}{{\text{l}}_{\text{2}}}\,+\,{\text{2N}}{{\text{H}}_{\text{4}}}{\text{OH}} \Rightarrow {\text{ Zn }}{\left( {{\text{OH}}} \right)_{\text{2}}}+{\text{2N}}{{\text{H}}_{\text{4}}}{\text{Cl}}$$

2 $${\text{Zn }}{\left( {{\text{OH}}} \right)_{\text{2}}} \Rightarrow {\text{ZnO}}\,+\,{{\text{H}}_{\text{2}}}{\text{O}}$$

#### Preparation of pure PMMA and PMMA/ZnO nanocomposites

The free radical emulsion polymerization process was used to synthesize the nanocomposites of PMMA/ZnO, where the nanoparticles were evenly embedded into the PMMA polymer matrix through mixing them with an MMA monomer solution, followed by a polymerization process. This leads to the production of highly homogeneous nanocomposites with superior mechanical strength and thermal stability^15^. The loaded percentage of ZnO NPs varied from 1 to 5 wt% of the MMA monomer. First, the calcinated ZnO NPs powder was homogenously dispersed in absolute ethanol using an ultrasonic cleanser processor with a 40 kHz operating frequency and 100 W power for 2 h. Then, the dispersed solution was put into a three-necked round-bottomed glass reactor with a reflux condenser, which was supplied with a magnetic stirrer, thermograph, and dropping tube. In order to help form micelles in the water and provide a high-yield polymer, a certain amount of surfactant SLS was added as an emulsifier^16^. The emulsifier can be sufficiently absorbed on the nano-ZnO hydrophobic surface when the solution is stirred at a high rate. After that, the reactor was filled with 10 ml of MMA monomer, which was introduced gradually by the dropping tube. Then, a calculated amount of KPS (initiator) was also added drop-wise into the reactor, while maintaining the reaction temperature at 75 ± 3 °C by circulating water in the reaction flask’s jacket. After 8 h of stirring, the temperature was gradually reduced to room temperature naturally, and the obtained nanocomposite emulsion was repeatedly centrifuged with absolute ethanol and distilled water to remove any surfactants in the sample. The yielded white precipitate was then dried at 60 °C to get rid of the unconverted monomer and moisture from the samples.

#### Film formation

To convert the synthesized PMMA/ZnO nanocomposite powder into a thin film, a calculated amount of the mixture nanocomposite was dissolved in toluene solvent. The solution was then cast into a Petri dish and dried at 60 °C for 10 h. Subsequently, the films can be obtained by rapidly cooling the Petri dish in cold water and removing the film. The thickness of the obtained films was measured using a high-precision digital micrometer with a range of 0.035–0.045 mm approximately.

#### Characterization techniques

In this study, several characterization techniques were used to analyze and investigate the structural features of ZnO NPs, pure PMMA, and PMMA/ZnO nanocomposites. Fourier-transform infrared spectroscopy (FTIR) was carried out using a Bruker FT-IR spectrometer (Invenio S, Germany) with the spectra in the range of 4000 to 400 cm^− 1^ wavenumber. Using transmission electron microscopy (TEM) with a JEOL-JEM-2100, Japan, at 200 kV, the average particle size of the synthesized ZnO particles could be measured. For crystalline/amorphous forms of the samples, an X-ray diffraction (XRD) scan was identified and performed by a Bruker D8 advance powder XRD, Germany, with a Cu Kα radiation source, with λ = 1.5405 Å, with a tube operating voltage of about 40 kV and current electron probe of 40 mA, and Bragg’s angle (2θ) extended from 5 to 80º. Additionally, for studying the thermal stability and degradation processes of the nanocomposites, thermograms were taken for each sample using the thermogravimetric analysis (TGA) instrument (SHIMADZU Testing & Weighing Equipment Division, KYOTO, JAPAN). TGA was performed under a nitrogen atmosphere with a constant flow rate at an increment of 20 mL per minute in the temperature range of 20–800 °C at a rate of 10 °C/min. For microstructure features and surface morphology, as well as the elemental composition of the polymer nanocomposite, a scanning electron microscope (SEM) and energy dispersive X-ray (EDX) with a model (JEOL JSM 6510 LV, Japan), operated at 30 kV, were used. Using Archimedes’ principle, the densities of the nanocomposite films with varying nanoparticle content were measured by the AS 220. R2 PLUS balance device instrument. Distilled water was used as the immersion fluid, measurements were taken at room temperature (17 °C), and a high-precision digital weighing balance was used to record the mass directly. Calculated density values for each specimen were obtained by dividing the mass of the sample in air by the weight loss in water (the difference between the readings of the mass before and after immersion in water)^17^. The hardness number was examined using the digital Vickers microhardness (Model: FM-7 Future-Tech. crop, Tokyo, Japan) technique at room temperature. Moreover, the mechanical properties of the samples were measured using an INSTRON (34SC-5) machine, encompassing Young’s modulus, tensile stress, and elongation at break. All measurements were performed in triplicate (*n* = 3) for each sample to calculate the mean value and standard deviation (mean ± SD).

#### Antibacterial and antimicrobial activity evaluation

For biological tests, the antibacterial and antimicrobial activities of the polymer nanocomposites were tested against gram-negative bacteria (*Escherichia coli)*, gram-positive bacteria (*Streptococcus mutans*), and *Candida albicans* fungus. The liquid discs were soaked in the Petri dishes containing nutrient agar media seeded with the tested bacteria or fungi. The inhibition zones were recorded after 24 h at 38 °C of incubation.

## Results and discussion

### Structural analysis

#### Fourier transform infrared (FTIR) analysis

**Fig. 1:**
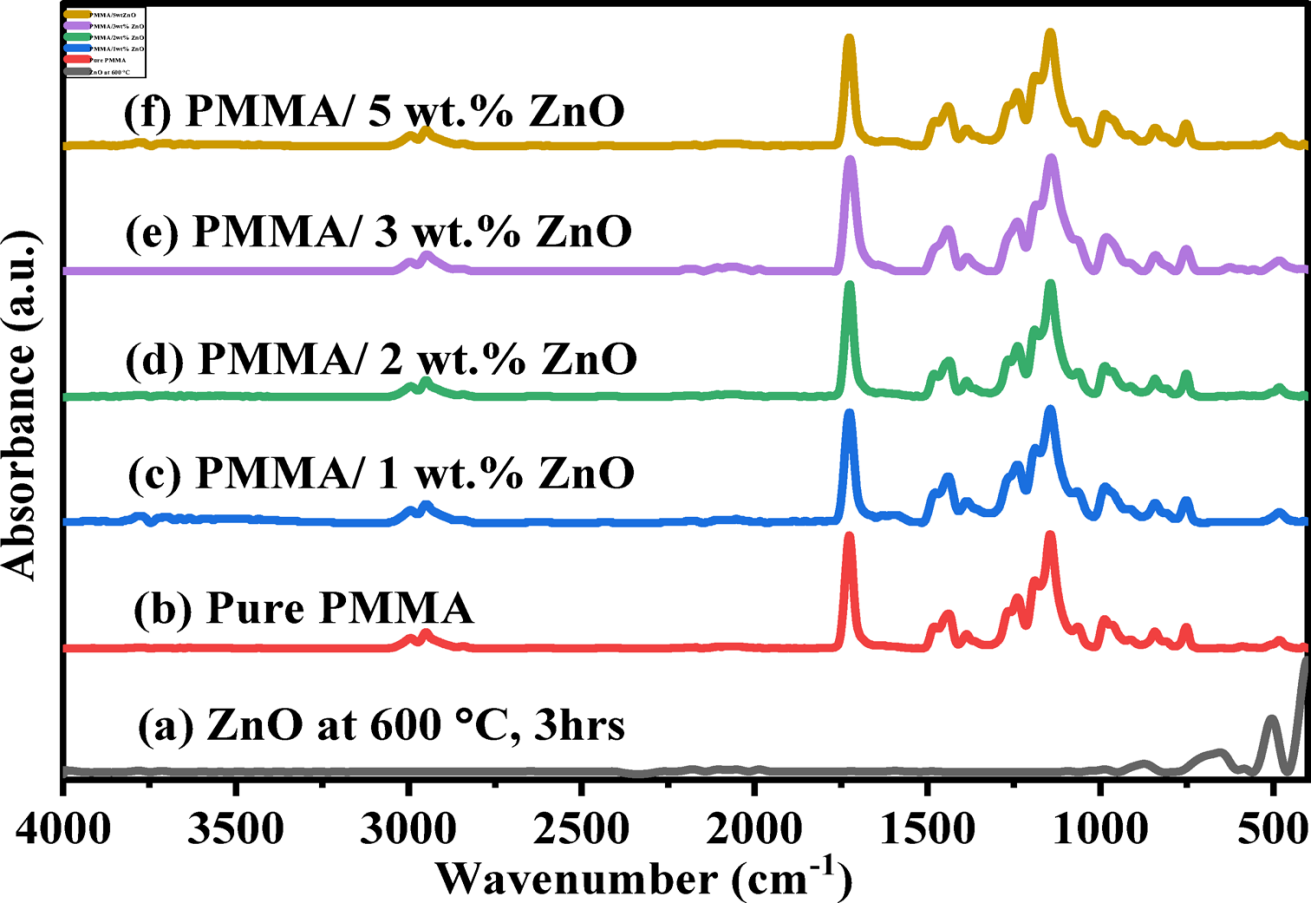
FTIR spectra of (a) ZnO NPs, (b) pure PMMA, and (c-f) PMMA/ZnO nanocomposites with different ZnO NPs concentrations of 1, 2, 3, and 5 wt%, respectively.

The functional groups of the prepared ZnO NPs and PMMA/ZnO nanocomposites were identified using FT-IR spectroscopy at room temperature, as shown in Fig. 1. For calcinated ZnO NPs at 600 °C for 3 h (Fig. 1a), a broad absorption band appeared in the range 3400–3550 cm^− 1^. This band is assigned to the stretching vibration of hydroxyl groups (O–H) on the surface of ZnO NPs, probably due to atmospheric moisture^18^. Additionally, the bands at 1600 and 600 cm^− 1^ are matching to the ZnO NPs bending vibration of both water molecules and the hydroxyl group in Zn (OH)_2_, respectively^18–22^. Moreover, the Zn-O vibrational bond was observed, as evidenced by the peak below 500 cm^− 1^^18,22^.

Figure 1b exhibited a typical strong stretching vibration peak at about 1726 cm^− 1^ that belonged to the ester carbonyl group (C = O), which is sensitive to coordination with metal oxides. This peak showed a slight broadening and shift towards a shorter wavenumber with increasing ZnO concentration relative to the pure PMMA. This can be explained as PMMA can create delocalized Q bonds by coordinating with Zn^2+^ ions on the ZnO particle surface^18,23,24^. Furthermore, those at ~ 1242 and ~ 1266 cm^− 1^ reflect the C-C-O stretch coupled with the CO stretch in the PMMA polymer matrix^18,23–25^. In addition, the absorption peaks observed at 2950, 2996, and 1384 cm^− 1^ were induced by the stretching vibration bands of C-H bonds (CH_2_ & CH_3_ groups)^18,23,25^, where their intensities slightly increased with the n-ZnO content. Bands at 987 and 749 cm^− 1^ were found by C-H bending^23^, whereas both bending and stretching vibrations of C = H and C = C appeared between 1330 and 740 cm^− 1^, respectively^25^.

FTIR studies were also employed to identify the natural state of ZnO NPs integrated into the PMMA polymer matrix, as well as any potential interactions. It was known that the change in the peak’s intensities and any shift in the original position of the FTIR spectra (even if slight changes) could be due to the intermolecular bonding between the PMMA polymer matrix and ZnO NPs^26^, which was attributed to the successful incorporation of ZnO NPs into the polymer matrix. As shown in Fig. 1c-f, the spectra of PMMA/ZnO NPs nanocomposites are nearly similar to the PMMA spectrum with slight changes upon ZnO NPs addition, due to the limited concentrations of these nanoparticles in the enlarged host matrix. This may cause a weak interaction between PMMA and metal oxide nanoparticles (e.g., hydrogen bonds, dipole–dipole interaction, or van der Waals, etc.).

#### X-ray diffractometer (XRD) analysis

**Fig. 2:**
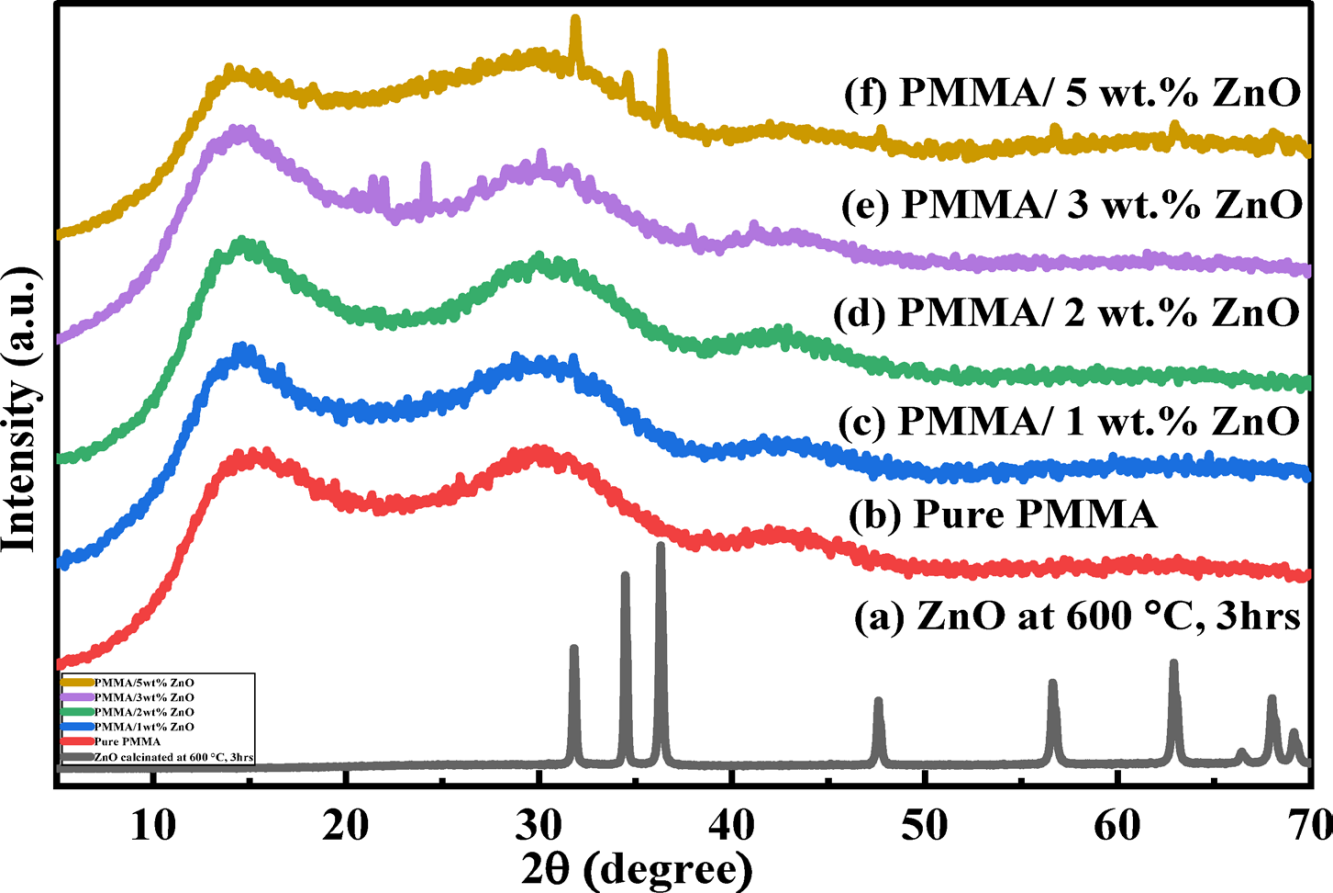
XRD spectra of the (a) calcinated ZnO NPs at 600 °C for 3 h, (b) pure PMMA, and (c-f) PMMA/ZnO nanocomposites loaded with different ratios of ZnO ranging from 1 to 5 wt%, respectively.

Figure 2 illustrates the X-ray diffraction pattern of (a) calcinated ZnO NPs, (b) pure PMMA, and (c-f) PMMA/ZnO nanocomposites with different ZnO NPs loadings, 1–5 wt%, respectively. As shown in Fig. 2a, XRD examination confirmed the presence of the crystal phase in the nanopowder, with no peaks from other phases, indicating the production of pure zinc oxide powder with a hexagonal wurtzite phase. The sharp and strong diffraction peaks with noticeable intensity reflect the high crystallinity of ZnO NPs with no sign of impurities. The characteristic peaks of ZnO have 2θ at 31.81º, 34.50º, 36.18º, 47.61º, 56.67º, 62.97º, and 68.08º, corresponding to the (100), (002), (101), (102), (110), (103), and (112) planes of the crystal lattice, respectively^19,21,27,28^, which well match with the Portable Document Format (PDF), 01–086-3978. Using the Scherrer equation, the average crystallite size (D) of the powder is determined as follows:

3 $${\text{D}}=({\text{K}}\lambda )/(\beta {\text{cos}}\theta )$$

where θ is the Bragg diffraction angle, β is the full width at half-maximum (FWHM) of the selected diffraction peak, k is a constant that depends on the shape of the crystallite (for spherically shaped crystals, it equals 0.9), and λ is the wavelength of the used X-ray. The calculated average value of the crystallite size of the calcinated ZnO NPs at 600 °C for 3 h was around 33.54 nm.

As shown in Fig. 2b, the X-ray diffraction pattern of pure PMMA includes a broad band (hump) at 2θ = 14.66º, which is characteristic of the amorphous nature of PMMA due to the bulky pendant ester group in its chains, as well as two centered shoulders at 30.84º and 43.19º^1,29–32^. Figure 2c-f illustrates PMMA/ZnO nanocomposites with different concentrations of ZnO NPs (1–5 wt%), respectively. The intensity of the nanocomposite peaks was gradually decreasing and widening compared to the pure PMMA, resulting in a crystallite size decrease^11^. Furthermore, the crystallinity of PMMA can’t be highly influenced by the increase in the weight% of ZnO nanofillers in the nanocomposites^29^. It is observed that no significant peaks related to the ZnO crystalline phase are present for samples in Fig. 2c-d, which demonstrates the successful dispersion of ZnO NPs at low content and formation of uniform grains of the polymer nanocomposites^29,33^. For the 5 wt% nanocomposite sample (Fig. 2f), the weak diffraction peaks for the (002) and (101) planes of the hexagonal wurtzite structure of ZnO appeared, respectively. This may be considered due to the little precipitated quantity of the nanofiller in the host polymer matrix^9^. Furthermore, it confirms that neither the ZnO nanofiller structure nor the orientation of the PMMA chains was influenced during the preparation process of the nanocomposites^1,30,34^.

#### Transmission electron microscopy (TEM)

**Fig. 3:**
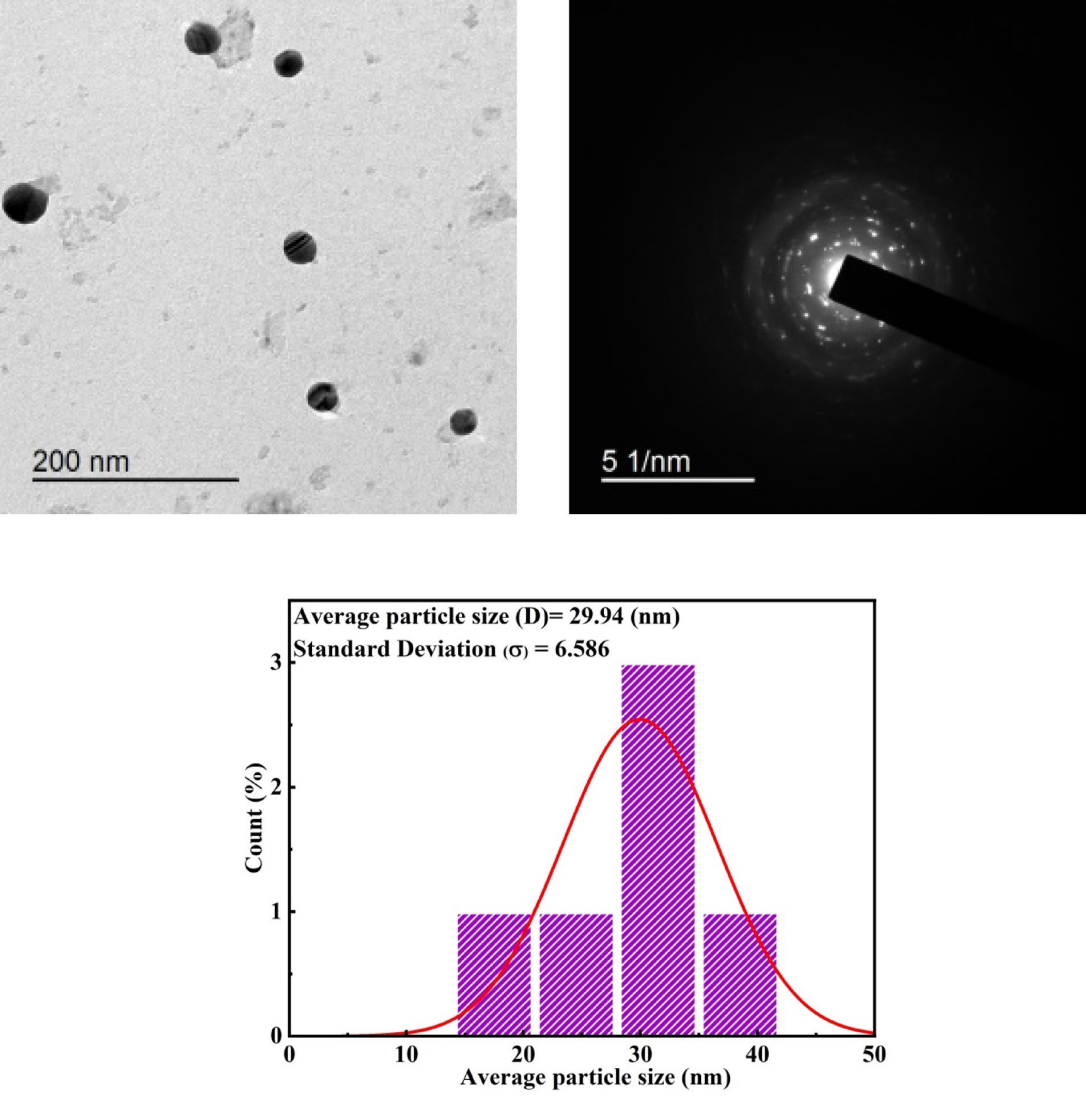
TEM images of the calcinated ZnO NPs at 600 °C for 3 h, electron diffraction, and histogram of particle size distribution of ZnO NPs.

One of the most widely used methods for exploring the shape and size of nanoparticles is transmission electron microscopy (TEM). A micrograph of calcinated ZnO NPs at 600 °C for 3 h has been illustrated in Fig. 3a. It is observed that most of the particles and crystals in ZnO are spherical in shape. The irregularity in the particle shape is considered due to the coalescence of grains during the calcination process^35^. The electron diffraction of ZnO NPs, as in Fig. 3b, shows that the particles were aligned to form a crystal structure, which has been previously confirmed by XRD data. Using the TEM micrographs, the particle size distribution histogram of ZnO NPs (Fig. 3c) shows nanocomposites having a size distribution of the particles with an average diameter of approximately 29.94 nm, which is in close agreement with that obtained from XRD investigation (33.54 nm). The powder agglomeration may explain the variance in particle size in TEM and XRD data, possibly related to the high surface energy of ZnO NPs. Thus, these agglomerates play an important role in influencing the longevity of polymer nanocomposites.

#### Scanning electron microscope (SEM)

**Fig. 4:**
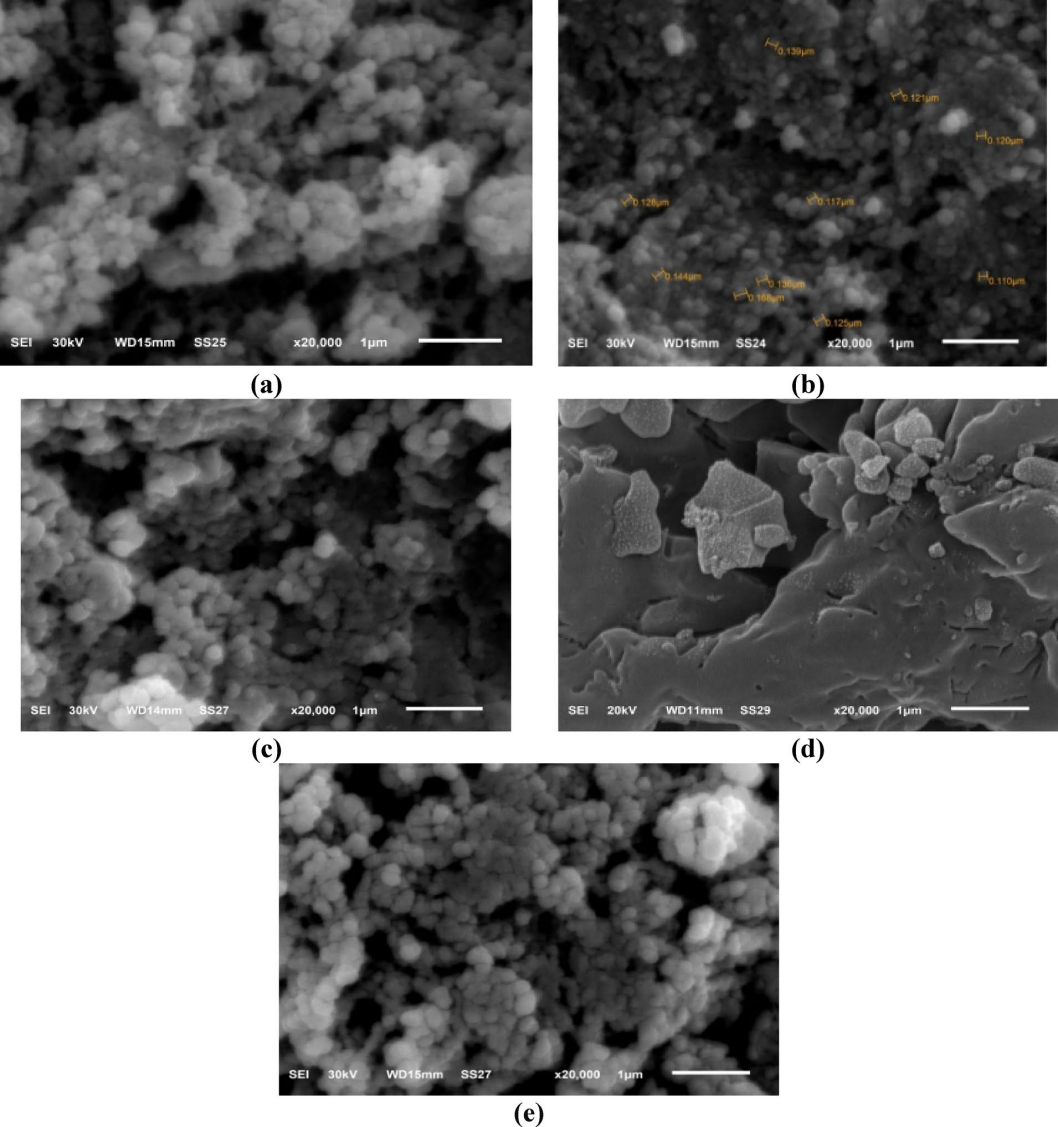
SEM images of (a) pure PMMA and (b-e) PMMA/ZnO nanocomposites loaded with different ratios of ZnO NPs, 1–5 wt%, respectively.

Scanning electron microscopy (SEM) was used to determine the morphology and the size of the examined samples, as well as the nanoparticle’s distribution within the polymer chain during the polymerization process. Figure 4a-e shows the SEM micrographs for pure PMMA and PMMA/ZnO nanocomposites with different concentrations of ZnO NPs. It is observed from Fig. 4a that the surface of pure PMMA was smooth and had various shapes of particles^36^. For PMMA/ZnO nanocomposites, a successful and well-dispersed ZnO NPs into the polymer chain structure was considered, as seen in Fig. 4b–e, resulting in a (reduced)/change in particle size^7^. Also, it is noted that the surface morphology of the polymer nanocomposites contains irregular white spots with a spherical-like shape dispersed throughout the polymer matrix up to 3 wt% ZnO NPs without observed agglomeration. For high concentrations of ZnO NPs (5 wt%), it is observed that the distribution of the nanofiller was not uniform in the polymer matrix, causing the n-ZnO to aggregate slightly due to the high surface energy of the nanoparticles^7,24,37^, which may lead to weak chemical bonds^38^. These aggregations strongly affect the structural and thermal properties of the nanocomposites^7,39^.

##### Energy dispersive X-ray (EDX) analysis

**Fig. 5:**
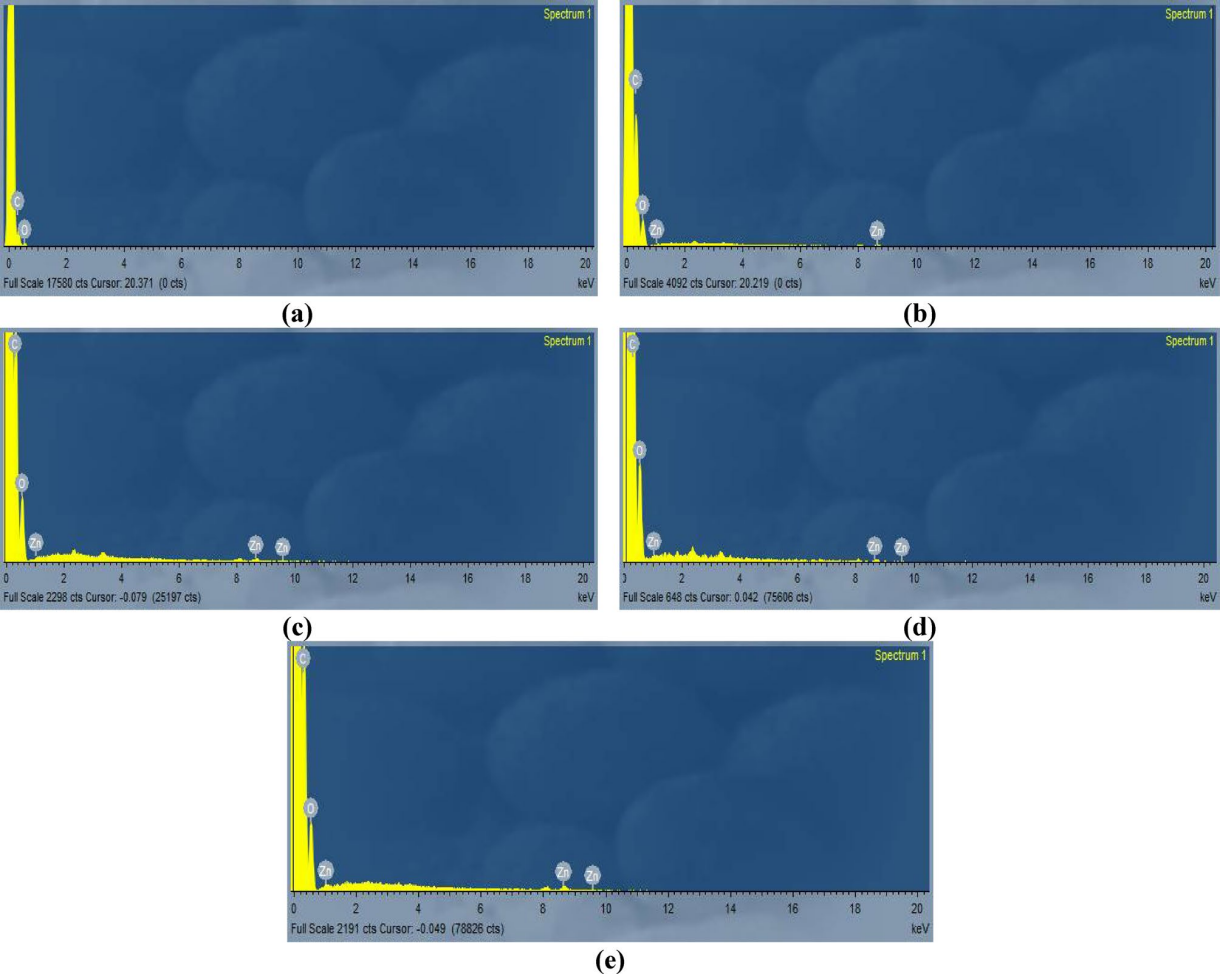
EDX images of (a) pure PMMA and (b-e) PMMA/ZnO nanocomposites loaded with different ratios of ZnO NPs, 1–5 wt%, respectively.

To determine the elemental composition and distribution of the synthesized polymer nanocomposites, an EDX analysis was performed. Figure 5 reveals the spectral analysis of EDX spectra with the chemical composition of PMMA and PMMA/ZnO nanocomposites by varying ZnO concentration. There are two characteristic elements of the PMMA polymer and PMMA/ZnO nanocomposites, which are represented by two intense peaks of C and O in all spectra due to the main backbone (CH_3_ & CH_2_) of PMMA^7,25,40,41^, as clearly explained in the FTIR section. The weight percentages of C, O, and Zn atoms were reported in Table 1. Figure 5b-e shows a new, less intense peak of the Zn element, which is attributed to ZnO NPs. Moreover, the O peak is a contribution between PMMA and ZnO^7,40^. Therefore, the EDX analysis confirmed the successful incorporation of ZnO into the PMMA polymeric chain and formation of polymer nanocomposites^25,40^. For the sample in Fig. 5e, there was a decrease in Zn (wt%) due to major clustering of the ZnO NPs in the PMMA matrix, which could be a result of the material porosity and tendency for occupying the natural niches by ZnO particles or due to water on the high surface of ZnO NPs.

**Table 1:** EDX composition of PMMA and PMMA/ZnO nanocomposites with 1–5 wt% ZnO.

Sample	Elements	Weight (%)	Atomic (%)
PMMA	Carbon (C), K Oxygen (O), K	72.09 27.91	77.48 22.52
PMMA/1 w.t% ZnO	Carbon (C), K Oxygen (O) K Zinc (Zn), K	73.61 26.22 0.17	78.87 21.10 0.03
PMMA/2 wt% ZnO	Carbon (C), K Oxygen (O) K Zinc (Zn), K	71.28 28.46 0.26	76.90 23.05 0.05
PMMA/3 wt% ZnO	Carbon (C), K Oxygen (O) K Zinc (Zn), K	71.75 27.69 0.56	77.45 22.44 0.11
PMMA/5 wt% ZnO	Carbon (C), K Oxygen (O) K Zinc (Zn), K	71.80 27.82 0.38	77.40 22.52 0.08
Total	100		

K = Spectroscopic term related to EDX.

##### Surface roughness

**Fig. 6:**
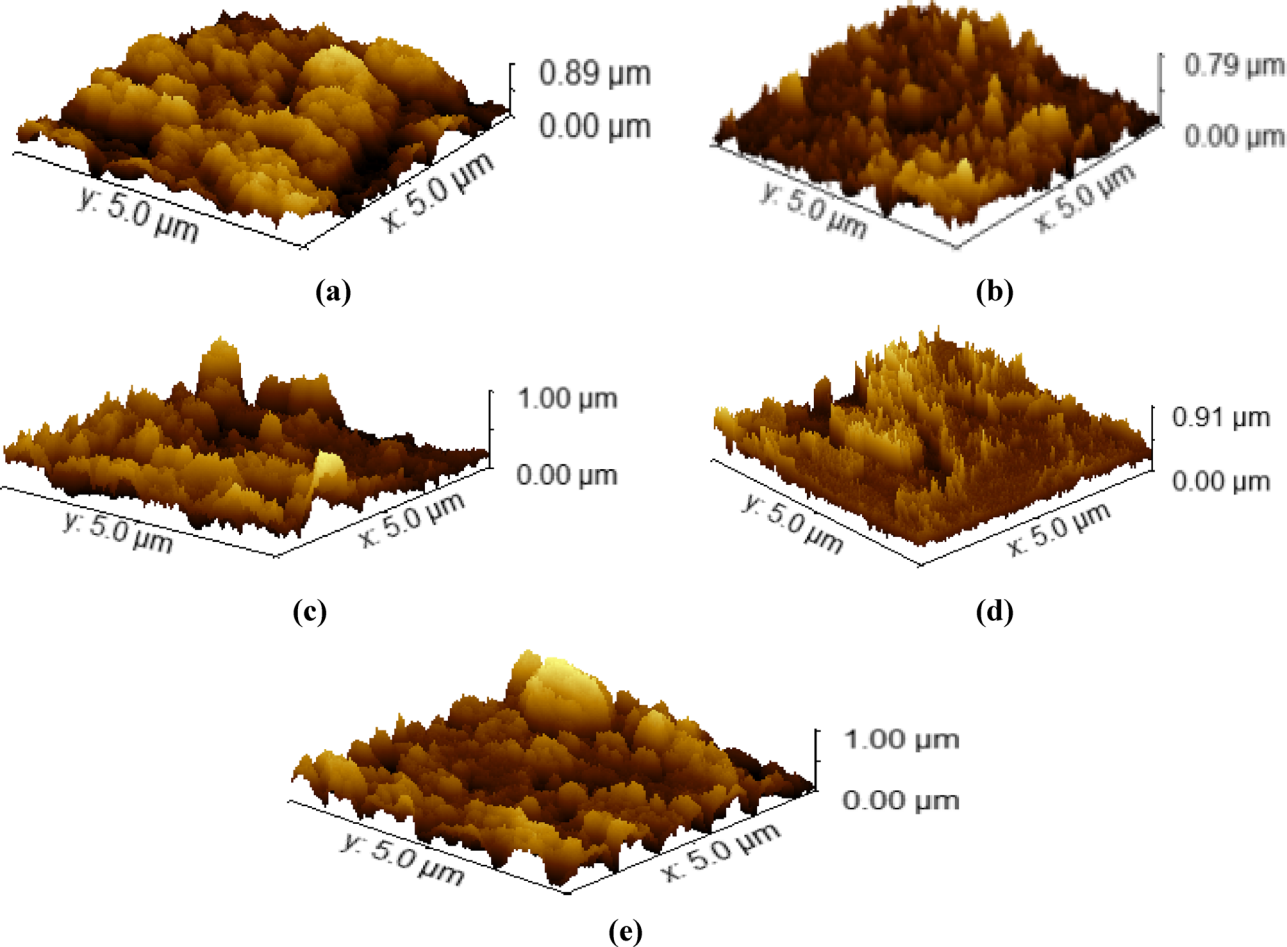
Typical areal (3D surface profilometric) roughness graphs of (a) pristine PMMA and (b-e) PMMA/ZnO nanocomposites loaded with different ratios of ZnO NPs 1–5 wt%, respectively.

The surface roughness can be extracted from SEM micrographs by using Gwyddion software for the statistical analysis of nanoparticles. The roughness parameters are studied as a function of ZnO NPs concentration in the host polymer matrix^9^. There are many different roughness parameters in use. The profile roughness parameters (Ra roughness average, R_q_ root mean square roughness) are more common^7^. The average maximum height of the profile (Rz) and skewness (Rsk) are other common parameters used. As the ZnO nanoparticle’s weight% increased, the surface became rougher to a certain extent. At larger ZnO loadings (5 wt%), the small ZnO particles (white spots) start to agglomerate, indicating ZnO segregation into the polymer matrix. Table 2 contains the Ra and R_q_ values for pure PMMA and PMMA/ZnO nanocomposites. As can be noticed from the table, neat PMMA has the lowest ones (16.84, 21.86 nm), while the highest values were observed for 3 wt% of PMMA/ZnO nanocomposites (30.39, 38.75 nm), respectively. For 1 & 2 wt% PMMA/ZnO nanocomposites, the Roughness average (Ra) and Root mean square roughness (Rq) values are intermediate. It was observed that every small aggregation of the nanoparticles increases the roughness at the nanoscale^23^. Typical areal (3D surface profilometer) roughness graphs of pristine PMMA and various ZnO NP ratios in the composites are shown in Fig. 6. The micrographs of the nanocomposite exhibit increased surface roughness with increasing ZnO nanofiller ratio up to 3 wt% ZnO NPs and a decrease for high concentration, as illustrated in Table 2. Such changes refer to the chemical adhesion between the ZnO NPs and the PMMA polymer, as demonstrated by the FTIR data.

**Table 2:** Values of different surface roughness parameters in (nm).

Sample	Ra (nm)	Rq (nm)	Rz (nm)	Rsk (nm)
Neat PMMA	16.837	21.861	145.487	−0.0758
PMMA/1 wt% ZnO	17.518	22.509	162.495	0.1230
*PMMA/2 wt% ZnO*	***19.887***	***25.598***	***169.312***	***27.4680 × 10⁻³***
*PMMA/3 wt% ZnO*	***30.388***	***38.752***	***275.111***	***0.0635***
*PMMA/5 wt% ZnO*	***21.946***	***29.147***	***210.731***	***0.0532***

Where Ra is the roughness average, Rq is the root mean square roughness, Rz is the average maximum height of the profile, and Rsk is the skewness.

### Thermo gravimetric analysis (TGA& DTG)

**Fig. 7:**
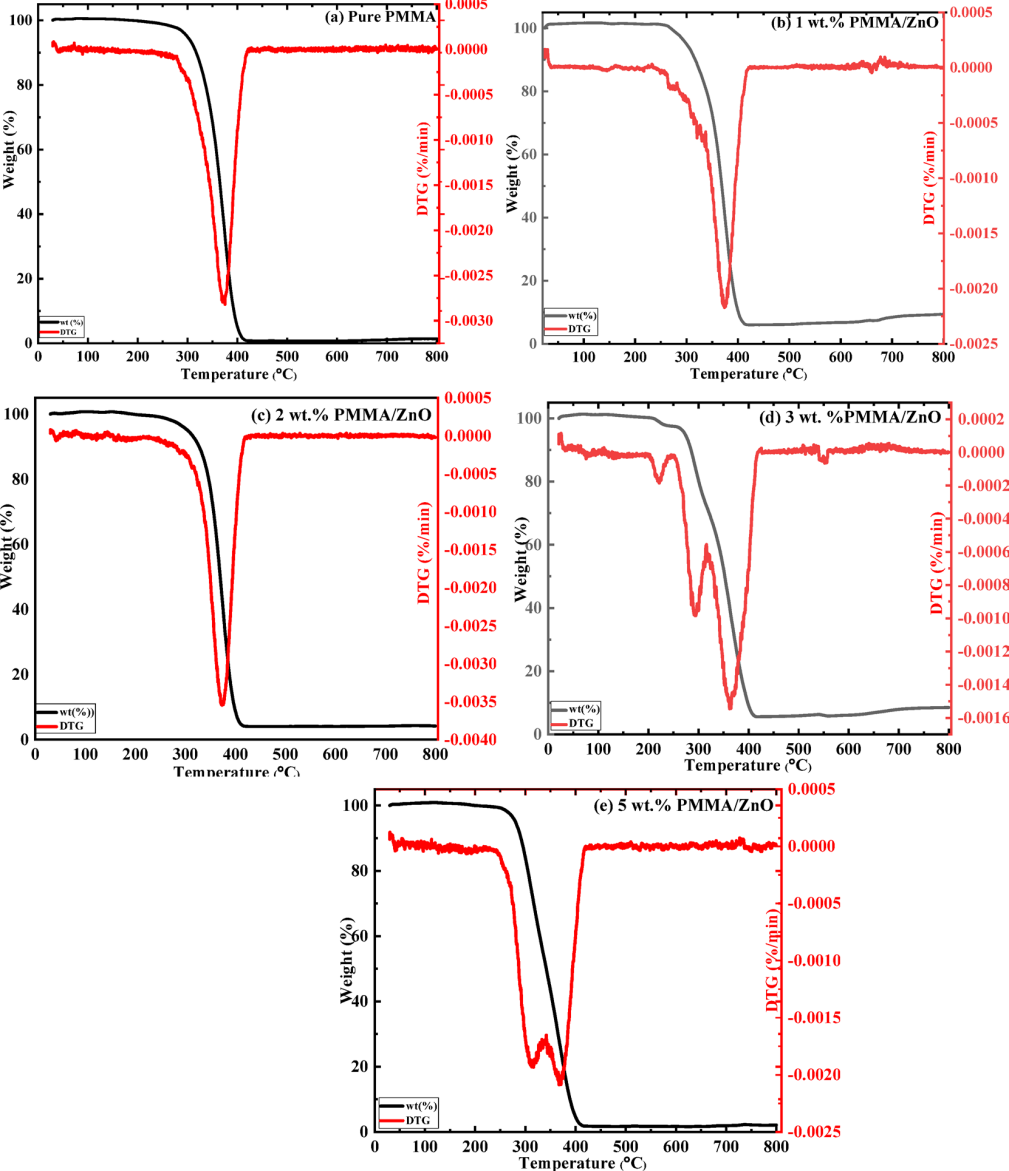
Thermogravimetric charts and DTG thermograms of (a) neat PMMA and (b-e) PMMA/ZnO nanocomposites with different ZnO NPs content (1–5 wt%), respectively.

One of the most common and widely used methods for determining the degradation temperature and the thermal stability of polymer nanocomposites is thermogravimetric analysis (TGA). Thermogravimetric analysis curves (TGA/DTG) were employed to study the various additions of the nanofiller effect on the thermal properties of the nanocomposites, as demonstrated in Fig. 7. Heating the nanocomposites occurred from room temperature (~ 25 °C) to 800 °C in the presence of nitrogen at a rate of 10 °C/min, where the weight loss was recorded as a function of temperature^42,43^. However, the improvement of the thermal properties of polymer composites was, to a certain extent, dependent on several factors, such as inorganic nanoparticle type, preparation method, composite structure, and the amount of the added nanofiller^40,44^. Pure PMMA has weak thermal properties^42^, but incorporation of ZnO NPs into the polymer matrix will act as a nucleating agent for heat absorption and enhance the thermal stability^9,24^. Figure 7a-e illustrates the TG thermogram of pure PMMA and PMMA/ZnO nanocomposites with different loadings of ZnO NPs (1–5 wt%). It is observed that for all prepared samples, there was a minor weight loss of about ~ 1.5–3.5% in a temperature range of ~ 30–300 °C, which was due to water desorption and initiator fragments^11^, as well as a depolymerization step, which originated at weak head-to-head linkages^7,26^.

Different stages of weight loss have been recognized according to different modes of initiation of depolymerization processes. For pure PMMA, it is observed that the only main degradation stage occurred through the temperature range of ~ 260–420 °C, where a complete decomposition is at nearly ~ 375 °C with a weight loss of ~ 98%. This refers to the degradation at which a random scission of the polymer backbone (carbonyl group) was initiated^9^. For 1 and 2 wt% ZnO addition, the degradation stage occurred between T = ~ 293–420 °C and 297–420 °C, resulting in a weight loss of about ~ 96.5% and 92.5%, respectively. The higher degradation temperature refers to the nanocomposites of PMMA/ZnO being higher in thermal stability than the neat PMMA, which was in good agreement with other groups^7,45^. At the final temperature (800 °C), the polymer matrix was nearly completely thermally degraded (Fig. 7a), where the residue on the TGA curves represented the estimated amount of the ZnO nanofiller present in the nanocomposites (Fig. 7b-e)^9^. Furthermore, the addition of 3 wt% ZnO into the polymer results in three stages of degradation: T = ~ 21–260 °C and ~ 259–336 °C and ~ 336–420 °C, with a weight loss of 2.7, 31.4, and 60.3 wt%, respectively. This means that the thermal properties of PMMA/ZnO nanocomposites increase with increasing concentration of ZnO NPs, i.e., the weight loss reduction from ~ 98% to 92% occurred for a 3 wt% ZnO NPs loading in the prepared nanocomposites (⁓ 6% thermal stability enhancement). This could be attributed to the barrier effect of well-dispersed nanoparticles, which restricts chain mobility and slows down thermal degradation pathways. Moreover, it was referred to the polymer matrix’s physicochemical bonding density strengthening^26^ and some interaction between the matrix and the nanofiller^9^, as previously demonstrated in FTIR analysis. However, at 5 wt% ZnO, there was a slight increase in the weight loss of ~ 94.7% through two stages of degradation. It can be demonstrated that there was an observed enhancement in thermal behaviors of the prepared nanocomposites compared to pure PMMA, but this improvement is to a certain extent due to the amount of the added nanofiller^44^. Thus, the improved thermal stability of the PMMA polymer matrix was up to 3 wt%, and more nanofiller additions result in agglomeration formation and possible phase separation, which can act as thermal defects, facilitating heat transfer and accelerating local decomposition, and causing a reverse improvement in the thermal behavior^37,39,46^.

Figure 7a-e illustrates also the DTG curves of pure PMMA and PMMA/ZnO nanocomposite loaded with different filler contents. It is clearly observed that a maximum thermal degradation temperature (T_max_) can be detected^47^. For pure PMMA, a complete decomposition was at ~ 400 °C, where the highest thermal decomposition (T_max_) was recorded at 372.5 °C. However, for PMMA/ZnO nanocomposites with 1 wt% and 2 wt% ZnO NPs, their T_max_ was observed at 376 °C and 374 °C, respectively. On the other hand, for 3 wt% PMMA/ZnO nanocomposites (optimum concentration), T_max_ was divided into two main peaks, which were located at nearly 293.8 and 363.3 °C. However, at high concentrations of ZnO NPs, the two peaks of T_max_ were located at ~ 315 and 370 °C.

### Density measurement

**Fig. 8:**
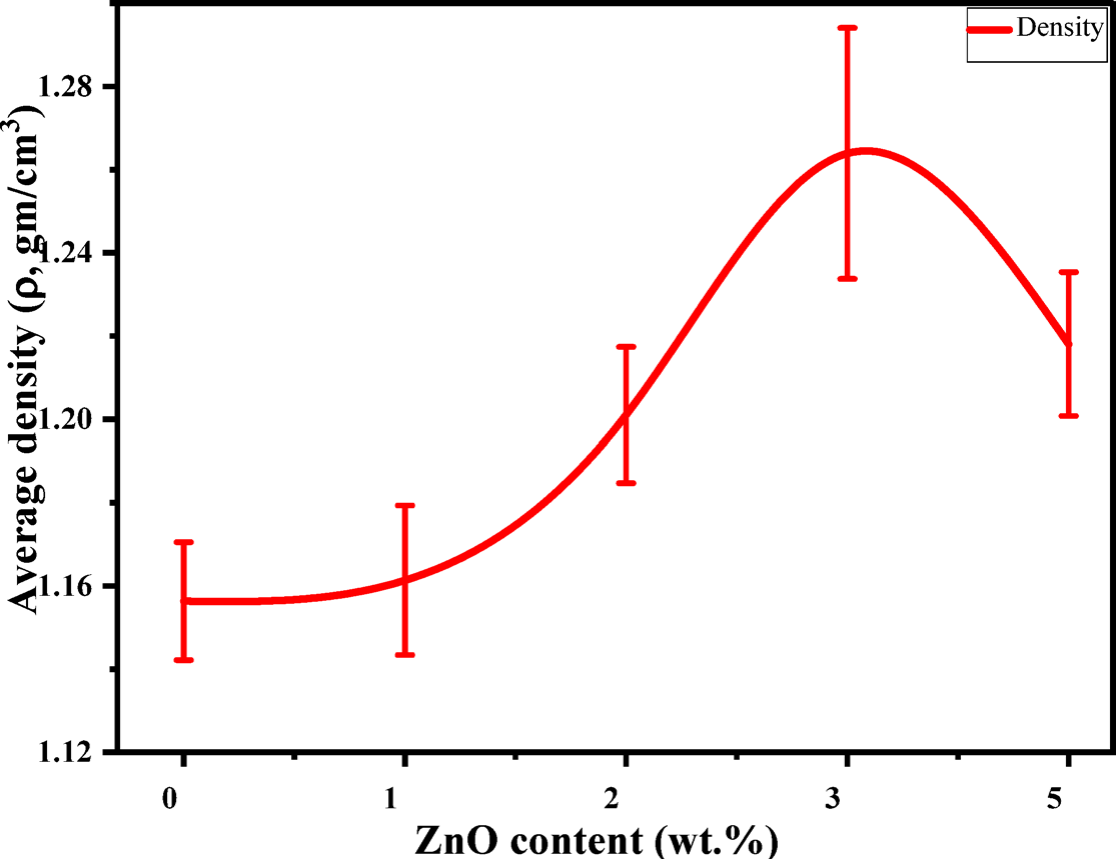
The average density of pure PMMA and PMMA/ZnO nanocomposites with varying ZnO NPs content (1–5 wt%), respectively.

Figure 8 shows the average density of pure PMMA and PMMA/ZnO nanocomposites with different ZnO NP concentrations (1–5 wt%). There was a positive correlation between density measurements and filler content in weight fractions of 1–3 wt%. This may refer to the prepared samples being dense and lower in porosity due to the greater density of ZnO (5.606 g/cm^3^) relative to PMMA (1.18 g/cm^3^)^17,48^. Therefore, this confirms that prepared nanocomposite samples are dense and have a liquid diffusion resistance with low porosities compared to the pure PMMA^17^. At higher ZnO NP content (> 3 wt%), the average densities showed a decreasing behavior. This trend leads to the suggestion that the sample containing 3 wt% is denser than that of other concentrations.

It can be noted that the increase in density values for the sample containing 3 wt% indicates a highly compact structure due to the well-dispersed ZnO through PMMA chains during the polymerization process, which can lead to minimizing the formation of voids and enhancing thermal stability and stiffness, as illustrated in TGA results. Nevertheless, the density begins to decrease at higher filler content, signifying the start of agglomeration formation of ZnO NPs. As a result, a decrease in the overall density and packing efficiency of the polymer-nanoparticle system occurred, causing micro-voids or defects within the matrix.

### Mechanical properties

#### Vickers micro-hardness test

**Fig. 9:**
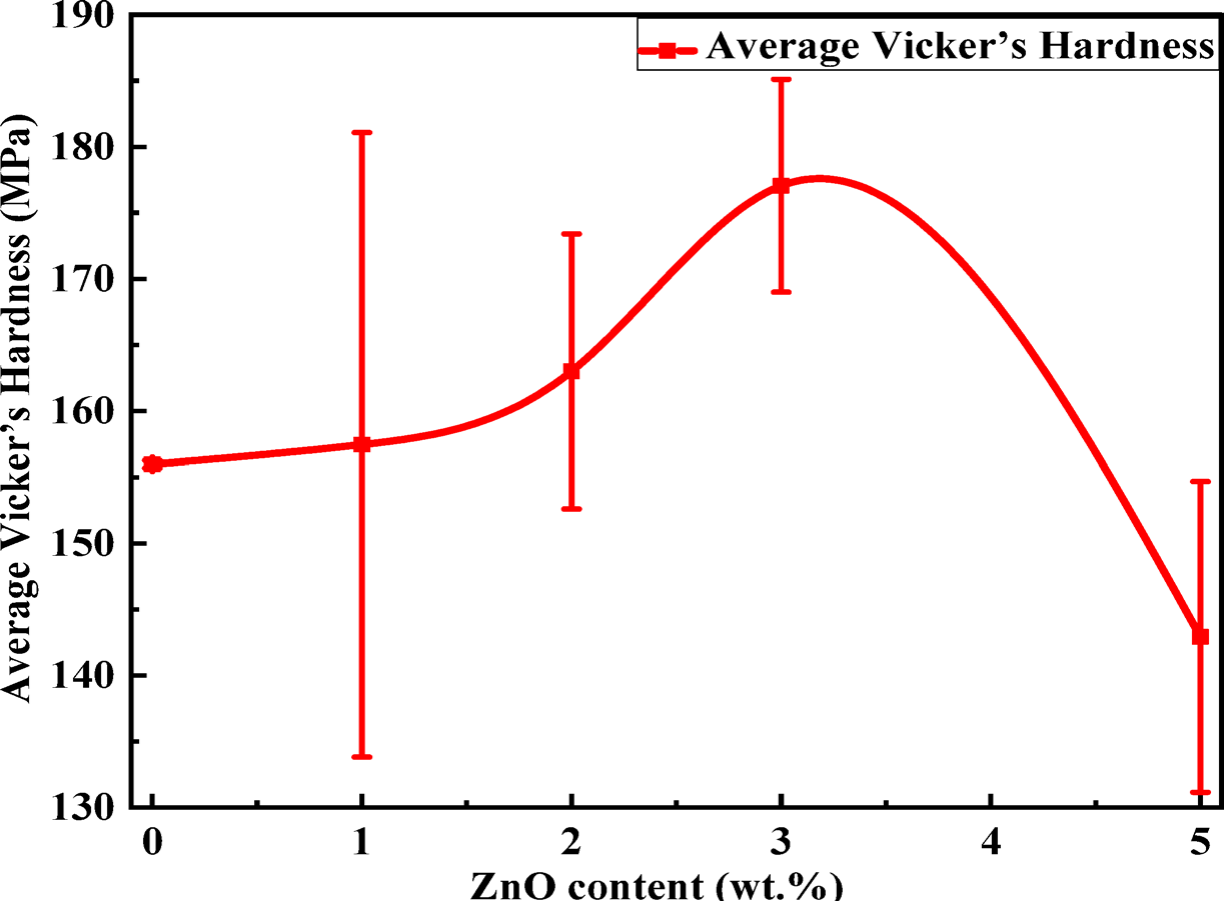
Hardness as a function of ZnO content for PMMA/ZnO nanocomposite films.

The hardness test measures the resistance of the sample against scratching and plastic deformation under an indentation load^1^. According to theoretical considerations, the addition of inorganic nanofiller particles to the polymer matrix increases the hardness of the material^49^. For instance, the effect of ZnO content on the hardness behavior of polymer nanocomposite films is investigated. The hardness number was examined using the digital Vickers microhardness (Model: FM-7 Future-Tech. crop, Tokyo, Japan) technique at room temperature. A given force of 10-gram force N was applied in a consistent manner for a required period of 5 s, which is appropriate for the measurement of semi-rigid and hard plastic materials. For each specimen, three indentations were made at randomly selected points. All measurements were performed in triplicate (*n* = 3) for each concentration to calculate the mean hardness value and standard deviation (mean ± SD), reported as the representative value for each one. Figure 9 illustrates the change in the hardness number of PMMA polymer modified by the different additions of ZnO NPs. The micro-hardness number (Hv) can be automatically calculated by using the following formula^50^:

4 $$\:\text{H}\text{v}=\frac{1.854\:\:F}{{d}^{2}}$$

where F = the applied load on the indenter, expressed in g-force, and d = the mean diagonal of indentation, in mm, and by using the values of d, the micro-hardness number (Hv) is evaluated in (gf/mm^2^). The average Vickers hardness data (HV) was obtained from 3 readings for each sample with three indentations at different areas for each sample for each concentration.

As shown in Fig. 9, the behavior of hardness with composition is seen to be similar to that of density changes. In general, the average hardness is also found to increase with increases in ZnO content to reach its maximum value at about 3 wt%, then decrease again with further addition of ZnO. The hardness number increases by ~ 13.5%, which is a statistically significant difference, which may be attributed to the good dispersion of the nanoparticles in the host polymeric matrix and good interfacial adhesion with the polymer chains^1^. This observed increase can be illustrated as the ZnO NPs, reinforcement effect, which transfers load from the PMMA matrix to the rigid filler phase. However, with further addition of ZnO NPs, agglomeration begins to occur and may even act as stress concentrators, causing a decrease in the hardness value. Therefore, from the discussion based on both density and hardness number, improvement in the properties of acrylic denture can be mainly considered through a certain addition of particulate fillers^1^. Therefore, the good dispersion of nanofillers was identified as a critical determinant for yielding a notable increase in PMMA mechanical performance^7,37,46^.

#### Tensile strength

**Fig. 10:**
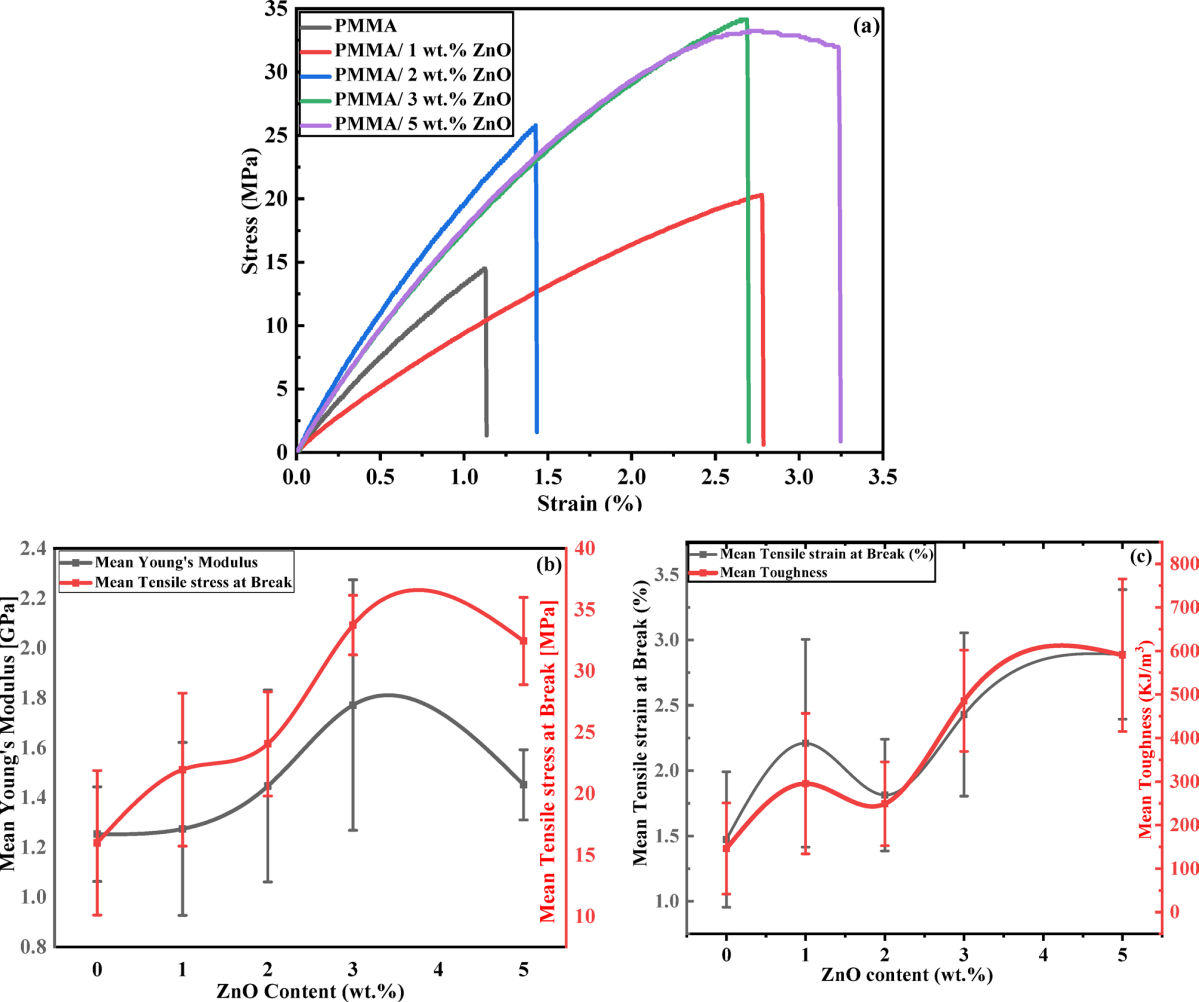
Mechanical properties of PMMA and PMMA/ZnO nanocomposites synthesized with varying loading of ZnO NPs: (a) Stress-strain curves, (b) tensile Strength and Young’s (or elasticity) modulus, and (c) percentage elongation, and toughness.

The mechanical properties, including Young’s modulus, tensile strength, and elongation at break, for pure PMMA and PMMA/ZnO nanocomposites were examined as in Fig. 10a. Tensile strength test results reveal that the PMMA has a minimum tensile strength average of ⁓ 16 MPa. For 3 wt% ZnO in the nanocomposites, a remarkable increase in the tensile strength was observed with an average of 33.7 MPa, which was an optimum concentration. This increase in the tensile strength results could be explained as a good dispersion of the particles into the polymer chain (as illustrated by SEM micrographs) and strong interfacial bonding between the two phases (i.e., H-bonds), resulting in an improvement in the crystallinity of the material^51–53^ (as reported in XRD data). Besides, the average tensile strength of 1, 2, and 5 wt% ZnO loading in the nanocomposite films was ~ 22, 24, and 32.5 MPa, respectively, as summarized in Table 3. In contrast, for higher loadings of ZnO NPs (5 wt%(, there was a slight reverse increase in the tensile strength. This could be due to the formation of weak spots in the matrix, which originated from the agglomeration/segregation and non-uniform distribution of the nanoparticles. Therefore, the formation of nanofiller aggregation yields a stress field concentrated around them, causing easy and rapid propagation of cracks. This could cause an interface debonding between the matrix and the particle, resulting in premature failure and reduction of the tensile strength, as mentioned in other works^51–53^.

#### Young’s modulus

Young’s modulus was a mechanical property that showed similar behavior and explanation as the tensile strength stated earlier for pure PMMA and PMMA/ZnO nanocomposites with different ZnO loadings. Figure 10c shows the results of the calculated Young’s modulus, which is optimum for 3 wt% ZnO loading in the nanocomposite with a 1.771 GPa value, which is much higher than the Young’s modulus of neat PMMA (1.253 GPa). On the other hand, for higher ZnO loadings (5 wt%), Young’s modulus begins to be slightly reduced (1.451 GPa), but it is still a larger value than the PMMA Young’s modulus. Table 3 summarizes the ultimate strength, toughness, and fracture strain values, which were obtained from the stress-strain relation. Briefly, the improvement in these mechanical properties is due to the interaction between the polymer matrix and the nanoparticles^54^.

**Table 3:** The mechanical properties of PMMA and PMMA/ZnO nanocomposites with different ZnO loadings.

Sample	*N* total	Ultimate strength [MPa]	% Elongation (% Fracture Strain)	Mean Young’s Modulus [GPa]	Mean Toughness KPa
Pure PMMA	3	15.992 ± 5.885	1.473 ± 0.519	1.253 ± 0.190	146.056 ± 104.630
PMMA/1 wt% ZnO	3	21.960 ± 6.236	2.21 ± 0.795	1.274 ± 0.347	295.213 ± 161.421
PMMA/2 wt% ZnO	3	24.063 ± 4.248	1.813 ± 0.428	1.446 ± 0.385	248.89 ± 96.417
PMMA/3 wt% ZnO	3	33.744 ± 2.429	2.43 ± 0.625	1.771 ± 0.503	485.596 ± 116.213
PMMA/5 wt% ZnO	3	32.450 ± 3.560	2.89 ± 0.496	1.451 ± 0.141	590.263 ± 175.140

#### Percentage elongation

Figure 10c shows that the increase is also in the percentage of elongation at break to 2.89% for 5 wt% ZnO, compared to pure PMMA (1.473%). It was observed that the percentage elongations for all nanocomposites have a marked rise, except for 2 wt% ZnO, which shows a slight inverse variation in percentage elongation. The improvement in the elongation percentage was due to the nanoparticles enhancing the polymer chain mobility and flexibility, even if they were not homogeneously dispersed, despite their rigid nature. In addition, the good interfacial bonding with the n-ZnO particles promotes the PMMA matrix to sustain larger deformation, yielding an improvement in elongation^52^.

### Antibacterial and antimicrobial activity evaluation

**Fig. 11:**
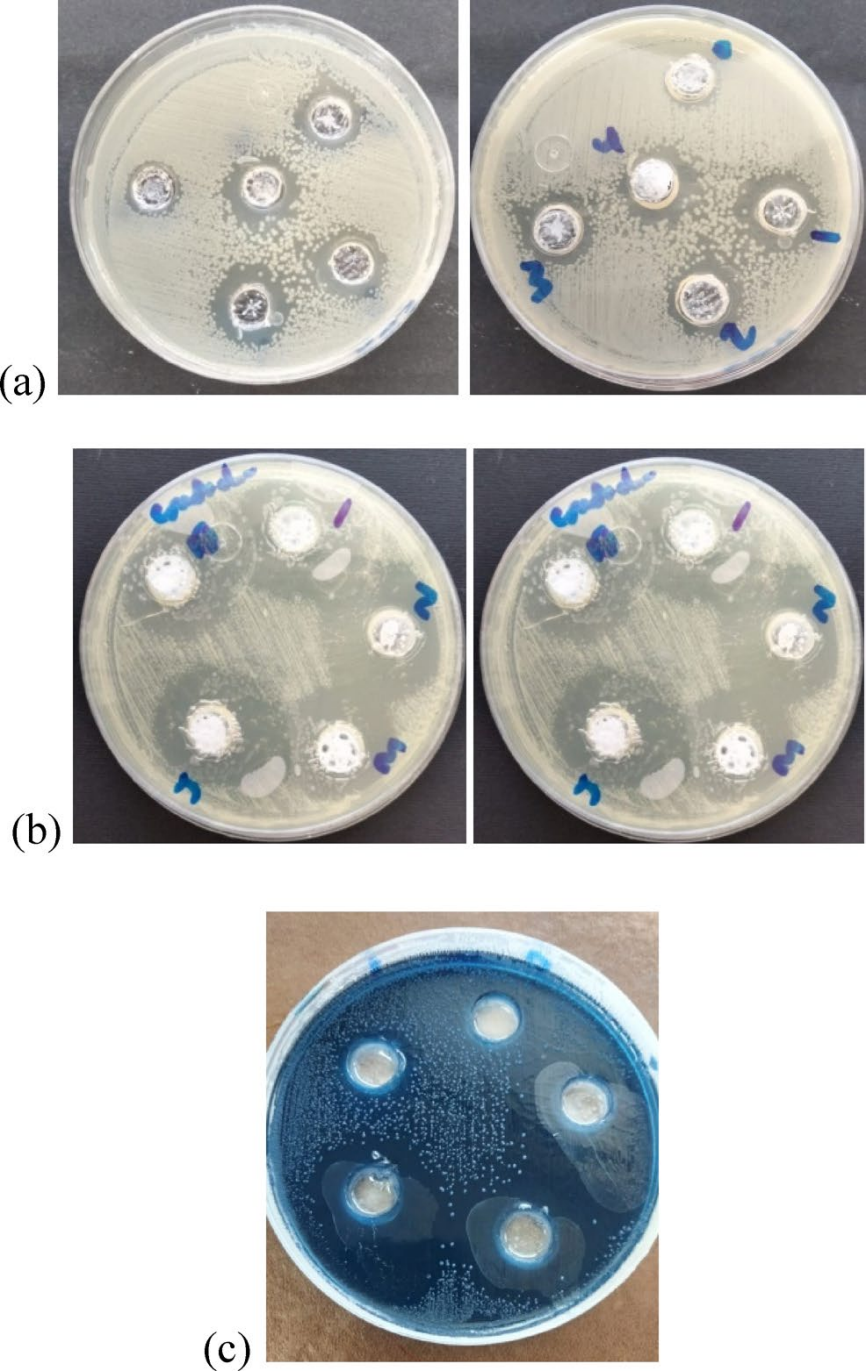
Images of antibacterial and antimicrobial behavior of pure PMMA and PMMA/ZnO nanocomposites for (a) *E. coli*, (b) *C. albicans*,* and (c) S. mutans*.

The antimicrobial agents (i.e., ZnO) are important to be added to the polymer matrix to improve the antibacterial capacity of these materials, due to their cariostatic effects^55–57^. Furthermore, the antibacterial activity of metal oxide NPs is dependent on various parameters such as particle size, surface area, morphology, concentration/dosage, the nature of the microorganisms, time of exposure to bacteria, etc. Some of the bacteria that cause dental caries are *Escherichia coli (E. coli)* (gram-negative), but the main cause is *Streptococcus mutans*. Therefore, the toxicity range of ZnO NPs is assessed against *E. coli* (gram-negative bacteria), *S. mutans* (gram-positive bacteria), and fungus (*Candida albicans*), as shown in Fig. 11a-c. PMMA is generally considered biologically inert and not intrinsically antimicrobial. The measured zones (15 mm for *E. coli* and 26 mm for *C. albicans*) are therefore more likely due to experimental artifacts, such as residual monomer, incomplete polymerization, or leaching of additives (Fig. 11a&b). According to the obtained results in Table 4, it can be found that ZnO NPs are effective antibacterial agents with increasing their content (1–3 wt%), through increasing the inhibition zone diameter^58–60^. They should also emphasize that optimal dispersion (3 wt%) maximizes the nanoparticle surface area, enhancing ion release and antimicrobial efficiency. However, ZnO NPs have a tendency to agglomerate (5 wt%), thus limiting the antibacterial activities^5^. The noticed inhibition zones were found to be small due to the low content of ZnO in the polymer matrix. The effective antibacterial ZnO agents can be explained based on the reactive oxygen species (ROS) released on the surface of these nanoparticles, such as hydroxyl radicals (OH^−^), superoxide (O^− 2^), and hydrogen peroxide (H_2_O_2_) that degrade the bacterial cell into CO_2_, H_2_O, and other non-toxic minerals. Since the hydroxyl radicals and superoxide are negatively charged particles, they cannot penetrate the cell membrane and must remain in direct contact with the outer surface of the bacteria; however, H_2_O_2_ can penetrate the cell. Also, Zn^2+^ ions can penetrate the bacterial cell, causing interruption of protein synthesis and producing toxic ROS, which causes DNA and cell membrane damage^61,62^.

**Table 4:** Inhibition zone values of the PMMA and PMMA/ZnO nanocomposites with different ratios of ZnO NPs.

Concentration in weight% (wt%)	E. coli	C. Albicans	S. mutans
	Diameter of inhibition zone (mm)	Diameter of inhibition zone (mm)	Diameter of inhibition zone (mm)
*Pure PMMA*	15	26	0
*PMMA/1 wt% ZnO*	17	27	18.667 ± 1.106
*PMMA/2 wt% ZnO*	19	31	25.833 ± 2.794
*PMMA/3 wt% ZnO*	20	34	29.667 ± 2.981
*PMMA/5 wt% ZnO*	20	30	23.6667 ± 1.247

## Conclusion

In the present work, the incorporation of the synthesized ZnO NPs, with different concentrations, into the PMMA polymer through the conventional free radical emulsion polymerization process, focusing on the structural, thermal, mechanical, and antibacterial properties of the yielded nanocomposites. XRD examination confirmed the successful formation of pure ZnO with a hexagonal wurtzite crystal phase, with an average particle size of about 33.54 nm, which was in close agreement with that obtained from TEM micrographs. FTIR, XRD, and SEM/EDX analyses confirmed the incorporation of ZnO NPs into the polymer. Thermogravimetric analysis (TGA/DTG) curves have shown a marked enhancement in the thermal stability of the nanocomposites via the weight loss reduction (~ 6% for 3 wt% ZnO NPs). The optimum concentration was found at 3 wt% ZnO NPs, resulting in an increase in density and average hardness number (~ 13.5% increase). These improvements were attributed to well-dispersed ZnO NPs, causing the stiffening and strengthening increase due to the physicochemical bonding in the polymer matrix. However, for 5 wt% ZnO NPs, there was a reduction in the thermal stability, density, and hardness measurements due to agglomerate formation. Moreover, the mechanical properties, including Young’s modulus, tensile strength, and elongation at break, for pure PMMA and PMMA/ZnO nanocomposites were examined. There was a remarkable increase in the tensile strength and Young’s modulus, which were observed with an average of around 34 MPa and 1.8 GPa for 3 wt% ZnO, which was an optimum concentration. It can be explained as the good dispersion of the particles into the polymer chain (as illustrated by SEM micrographs), strong interfacial bonding between the two phases (i.e., H-bonds), resulting in an improvement in the crystallinity of the material^51–53^ (as reported in XRD data). In contrast, for higher loadings of ZnO NPs (5 wt%(, there was a slight reverse improvement, which could be due to the formation of weak spots originating from the agglomeration/segregation and non-uniform distribution of the nanoparticles. Additionally, the inhibition zones are slightly increased (≤ 3 wt%) with increasing ZnO content for the antibacterial and antimicrobial behavior. In general conclusion, the study confirms that the ZnO NPs optimal concentration in the PMMA matrix is 3 wt%, at which the thermal and mechanical performances are highly significantly enhanced for producing advanced, favorable materials used in various dental applications, including denture base materials.

Although the 3 wt% ZnO composition exhibits promising characteristics, extending the study to a wider composition range (0.5–10 wt%) would provide a more comprehensive understanding of its structure and behavior impact on numerous features. Thus, future studies will extend that broader composition range to identify the more optimum concentration, assess possible threshold effects, and establish a more comprehensive understanding of the material’s performance and stability.

## Data Availability

The datasets used and/or analyzed during the current study are available from the corresponding author on reasonable request.
